# Hierarchical processing in the prefrontal cortex in a variety of cognitive domains

**DOI:** 10.3389/fnsys.2014.00223

**Published:** 2014-11-25

**Authors:** Hyeon-Ae Jeon

**Affiliations:** Department of Neuropsychology, Max Planck Institute for Human Cognitive and Brain SciencesLeipzig, Germany

**Keywords:** hierarchical processing, prefrontal cortex, BA44, language, visuo-spatial sequences, music, mental arithmetic, actions

## Abstract

This review scrutinizes several findings on human hierarchical processing within the prefrontal cortex (PFC) in diverse cognitive domains. Converging evidence from previous studies has shown that the PFC, specifically, BA44, may function as the essential region for hierarchical processing across the domains. In language fMRI studies, BA 44 was significantly activated for the hierarchical processing of center-embedded sentences and this pattern of activations was also observed in artificial grammar. The same pattern was observed in the visuo-spatial domain where BA44 was actively involved in the processing of hierarchy for the visual symbol. Musical syntax, which is the rule-based arrangement of musical sets, has also been construed as hierarchical processing as in the language domain such that the activation in BA44 was observed in a chord sequence paradigm. P600 ERP was also engendered during the processing of musical hierarchy. Along with a longstanding idea that a human’s number faculty is developed as a “by-product of language faculty”, BA44 was closely involved in hierarchical processing in mental arithmetic. This review extended its discussion of hierarchical “processing” to hierarchical “behavior”, that is, human action which has been referred to as being hierarchically composed. Several lesion and TMS studies supported the involvement of BA44 for hierarchical processing in the action domain. Lastly, the hierarchical organization of cognitive controls was discussed within the PFC, forming a cascade of top-down hierarchical processes operating along a posterior-to-anterior axis of the lateral PFC including BA44 within the network. It is proposed that PFC is actively involved in different forms of hierarchical processing and specifically BA44 may play an integral role in the process. Taking levels of proficiency and subcortical areas into consideration may provide further insight into the functional role of BA44 for hierarchical processing.

## Introduction

*Hierarchical processing* indicates that the process in the superordinate level controls, modifies, and modulates the process in the subordinate level operating over a longer period of time (Welford, 1951; Miller et al., 1960). Human cognitive architecture is generally known as a complex system composed of substructures as in hierarchical processing. Newell mentioned that “the human architecture is built up of a hierarchy of multiple system levels and it cannot be otherwise structured” (Newell, 1994, p. 117). The concept of hierarchical processing was deepened with a focus on the action domain denoting that human action is also hierarchically organized (Lashley, 1951). This notion was further explored by a line of research suggesting that an action is comprised of consecutive sub-sequences with “limited short-term goals” at multiple and hierarchical levels, in succession, resulting in “larger and longer units” within a hierarchical model (Fuster, 1989). This idea originated with the aim of elucidating hierarchical processing in the action domain and was then further extended to other domains such as language and music with the implication that linguistic and musical behaviors were inherited with features from motor planning and action (Lashley, 1951). Broadbent (1977) mainly predisposed the term “hierarchy” to describe the process by exemplifying diverse hierarchical structure in perceptual analysis, language, computer problem solving, and structuring in long-term memory. For example, in the language domain, the on-line build-up of grammatical structures (i.e., syntactic processing) is operated in a hierarchical way on the long-term structural knowledge in language (i.e., syntactic knowledge) (Patel, 2003).

Along with the development of cognitive neuroscience, studies about the neural mechanism underlying hierarchical processing have been proliferated, and as a consequence the dorsolateral prefrontal cortex (PFC) has been known to be the critical brain structure. One of the major roles of the PFC is to bind short-term goals to make larger and longer units with longer-term objectives. Therefore time is a critical quality for the PFC to be actively involved in traversing temporal discontinuities between short-term goals, which is called “cross-temporal contingencies” (Fuster, 1989, 2001). Interestingly, this temporal contingency is one of the key features of hierarchical processing. The overarching aspect of hierarchical processing is a temporal integration or temporal “schema” being related to an overall goal such that it should be imposed for a longer period of time in a complex situation where various subgoals are arrayed at multiple levels, that is, the hierarchical system (Lashley, 1951). Fitch and Martins (2014), distinguishing a hierarchical sequence from a hierarchical set, also acknowledged that a temporal order is compulsory for hierarchical processing. Consequently, cross-temporal contingencies function as the medium that connects hierarchical processing and the PFC.

According to a number of neuroimaging studies on hierarchical processing, varying areas of the PFC were activated depending on the diversity of cognitive domains, experimental tasks, and within- or between-group designs. Therefore, the aim of this article is to review the studies with neuroimaging techniques such as functional magnetic resonance imaging (fMRI), event-related brain potential (ERP), transcranial magnetic stimulation (TMS), near-infrared spectroscopy, magnetoencepholography (MEG), and with lesions in patients, emphasizing the neural underpinnings involved in hierarchical processing within the PFC in various cognitive domains such as language, visuo-spatial sequences, music, mental arithmetic, and action along with cognitive controls. In particular, Brodmann area (BA) 44 (pars opercularis) corresponding to the posterior part of Broca’s region on the inferior frontal gyrus will be mainly discussed in terms of hierarchical processing. This region has been known to support hierarchical processing in human language as a form of recursion as well as in broad cognitive domains as neural underpinnings for the manipulation of hierarchical structures (Hauser et al., 2002; Boeckx et al., 2014).

## Hierarchical processing in language

It has been suggested that language is characterized by hierarchical phrase structures; noun and verb phrases are arranged within a clause in a hierarchical way rather than a linear order of words (Moro, 2000; Friederici et al., 2011). Hierarchical processing in the language domain, more specifically, in syntax, has been mainly studied with two types of manipulation, that is, word order and center-embedding. The hierarchy of the sentence processing is increased by switching the position of the subject and object in the manipulation of word order (Fodor, 1978; Chomsky, 1993) and by embedding subordinate clauses into a superordinate clause in the case of center-embedding (Miller and Isard, 1964; Chomsky, 2002). Between these two manipulations, the processing of the center-embedded sentence seems to better explain linguistic hierarchy because, as mentioned earlier, the mediation of temporal contingency is the critical factor for the processing of hierarchy which is well-implemented in the center-embedded sentence by assigning a thematic role and monitoring a predicate with an intervention of embedded clauses (Friederici et al., 2011; Jeon and Friederici, 2013).

**Figure 1:**
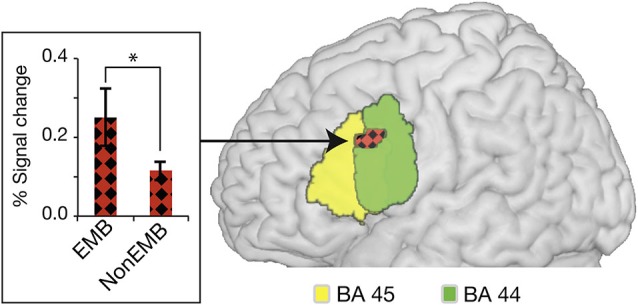
**Activation in BA44 for the processing of linguistic hierarchy in German**. The activation elicited by the processing of center-embedded sentences is overlaid on the cytoarchitectonic map of BA 44 (green) and BA 45 (yellow). Plot of the percent BOLD signal change from an activated cluster in the conditions is provided on the left; errors bars denote s.e.m. (*P < 0.01). EMB, center-embedded sentences; NonEMB, non-embedded sentences. (from Jeon and Friederici, 2013).

Much debate surrounds the processing of syntactic hierarchy in language, with Broca’s area, more specifically, BA44, at the center of the controversy. The dissociation has been shown in Broca’s area between the anterior and the posterior region with respect to hierarchical processing in the first language. Here, the first language is defined as the languages that people acquired during early childhood, approximately before the age of three years and learned with people who speak it (Saville-Troike, 2012). Some studies have obtained a strong activation in the posterior region of the inferior frontal gyrus (BA44) for the process of embedded clauses (Stromswold et al., 1996; Makuuchi et al., 2009; Santi and Grodzinsky, 2010; Jeon and Friederici, 2013). For example, when center-embedded sentences were compared with non-embedded sentences in German (Table 1 for stimulus examples), a strong activation was found in the posterior region of BA44 (pars-opercularis) (Figure 1; Jeon and Friederici, 2013). However, others have also found some of the activations in the anterior portion of the inferior frontal gyrus (BA45 and BA47). Studies using English as a test language found enhanced activations in left BA 44 and BA 45 extending to BA 47. This variance between the two languages may be dependent on a number of language-specific features for the processing of center-embedded structures. Languages with a relatively free word order such as German heavily depend on morpho-syntactic features whereas English, which has a relatively fixed word order, may be more dependent on the word position and semantic relation between the words (Friederici and Weissenborn, 2007). For example, a left anterior negativity (LAN) known as brain responses related to the processing of syntactic structures was elicited mostly in German but not often in English (Friederici and Weissenborn, 2007). Brain mapping studies also support the role of BA45 and 47 for the retrieval of semantic information and the processing of semantic relationships between words in syntactic hierarchy if the demand or stimulus configuration requires a considerable involvement of semantic processing (Friederici, 2002; Caplan et al., 2008; Newman et al., 2010). BA44, 45, and 47 have been also known to be actively involved when syntactic nodes (i.e., noun, verb, noun phrase, verb phrase, etc.) are unified in order to frame syntactic structures (Hagoort, 2005). This issue has already been discussed in several review articles in relation to syntactic complexity, syntactic ambiguity, or working memory (Grodzinsky, 2000; Bookheimer, 2002; Hagoort, 2005; Grodzinsky and Friederici, 2006; Rogalsky and Hickok, 2010; Friederici, 2011) and here I would like to point out that the particular function of BA44, BA45, and BA47 still needs to be specified across different experimental setups as well as various languages.

**Table 1:** Center-embedded and non-embedded sentences in German.

	Peter wusste, dass…
	*Peter knew that…*
EMB-S	
	der Schriftsteller, [der mit Uwe lebte], Andreas erkannt hatte.
	the writer_(Masc. Nom.)_, [who with Uwe lived], Andreas_(Acc.)_ recognized had.
	*the writer, who lived with Uwe, had recognized Andreas*.
EMB-O	
	den Schriftsteller, [der mit Uwe lebte], Andreas erkannt hatte.
	the writer_(Masc. Acc.)_, [who with Uwe lived], Andreas_(Nom.)_ recognized had.
	*Andreas had recognized the writer who lived with Uwe*.
NonEMB-S	
	der Dirigent die kleine Sarah und Anna besucht hatte.
	the conductor_(Masc. Nom.)_ the_(Fem. Acc.)_ little Sarah and Anna visited had.
	*the conductor had visited the little Sarah and Anna*.
NonEMB-O	
	den Dirigenten die kleine Sarah und Anna besucht hatte.
	the conductor_(Masc. Acc.)_ the_(Fem. Nom.)_ little Sarah and Anna visited had.
	*the little Sarah and Anna had visited the conductor*.

*Every sentence started with “Peter wusste, dass (Peter knew that)”. Stimulus examples for each condition are displayed above in German with a word-by-word translation in English underneath. Embedded structures for EMB-S and EMB-O are bracketed. EMB-S = Center-embedded structure, Subject first; EMB-O = Center-embedded structure, Object first; NonEMB-S = Nonembedded structure, Subject first; NonEMB-O = Nonembedded structure, Object first; Masc. = Masculine gender; Fem. = Feminine gender; Nom. = Nominative; Acc. = Accusative. (from Jeon and Friederici, 2013)*.

Several lesion studies have shown that atrophy of left Broca’s area is related to deficits in the processing of syntactic hierarchy (Gunawardena et al., 2010; Wilson et al., 2010b, 2012; Rogalski et al., 2011). For example, the patient group with nonfluent primary progressive aphasia having lesions in the pars opercularis (BA44) showed a similar degree of functional activations between the syntactically complex and simple sentences whereas the control group yielded more activations in the complex sentences than in the simple sentences (Wilson et al., 2010a). The role of the pars opercularis together with syntactic hierarchy was also supported by the study using voxel-based morphometry where the left pars opercularis was the only area predictive of low accuracy on syntactic processing in patients with nonfluent/agrammatic primary progressive aphasia (Deleon et al., 2012).

**Figure 2:**
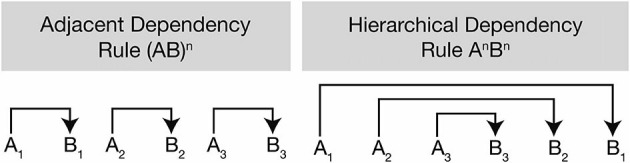
**Difference between adjacent dependency and hierarchical dependency rules**. The adjacent dependency rule is generated via the formula (AB)**^n^** by simple local transition between two items being positioned side by side (A_1_ to B_1_, A_2_ to B_2_, and A_3_ to B_3_). It involves the generation of sequences of alternating adjacent category pairs. The hierarchical dependency rule formulated by A*^n^*B*^n^* has more complex structures where two items (e.g., A_1_ and B_1_) are separated by embedded structures (e.g., A_2_ and B_2_, A_3_ and B_3)_ positioned between A_1_ and B_1_ (from Bahlmann et al., 2009).

Along with the syntactic hierarchy in natural language, the processing of hierarchical sequences in artificial grammar has also been studied because the intervention of semantic, phonological, or morphological processing can be avoided and all the critical variables can be easily controlled across the participants (Friederici, 2011). Two studies where center-embedded and non-embedded sentences were replaced with hierarchical dependency (i.e., A*^n^*B*^n^* rule) and adjacent dependency (i.e., [AB]*^n^* rule) (Figure 2) showed a main effect of hierarchy in BA44, suggesting that BA44 is recruited for the processing of hierarchical structures, be it in natural or artificial languages (Friederici et al., 2006; Bahlmann et al., 2008).

## Hierarchical processing in visuo-spatial sequences

It has been noted that BA44 is recruited in the processing of complex hierarchical structures in the visuo-spatial domain suggesting that this area may have a supramodal nature for dealing with a complex long-dependency structure. For example, abstract symbol sequences with adjacent and hierarchical dependency rules (Figure 2) were compared with each other for the study of hierarchical visuo-spatial sequence processing, resulting in higher blood-oxygen-level-dependent (BOLD) signal observed in BA44 for the condition of hierarchical dependency rule (Bahlmann et al., 2009). Likewise, Tettamanti et al. (2009) compared the processing of “word-based syntax” in the language domain with “symbol-based syntax” in the visuo-spatial domain. The Korean alphabet was used as visual symbols in the visuo-spatial domain in the participant group of native Italian speakers for the purpose of decreasing the possibility of employing undesirable strategies such as subvocalization or semantic association with common objects or entities. Two experimental conditions were recruited depending on whether the order of words and symbols was fixed (“rigid syntactic dependencies”) or not fixed (“non-rigid syntactic dependencies”); the former is never found in human languages whereas the latter represents “the core type of dependencies found in the syntax of all natural languages” (Tettamanti et al., 2009). Results showed that BA44 in the left hemisphere was activated only in the condition of non-rigid syntactic dependencies across the language and the visuo-spatial domains. In summary, both studies approached the neural underpinnings involved in hierarchical processing from the aspect of visuo-spatial domain by using visual symbols and discovered the involvement of BA44 for the process. This may implicate that BA44 is a supramodal area in the PFC as a core region for hierarchical processing regardless of the domains.

## Hierarchical processing in music

**Figure 3:**
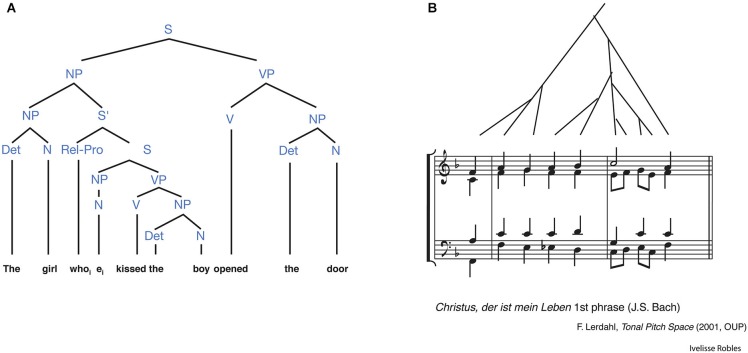
**Similarities in hierarchical structures between language and music. (A)** The hierarchical structure in an English sentence. This syntactic tree depicts the hierarchical relations among words and phrases. S, sentence; NP, noun phrase; VP, verb phrase; S^′^, sentence modifier [relative clause]; N, noun; V, verb; Det, determiner; Rel-Pro, relative pronoun. **(B)** A phrase from a composition by Johann Sebastian Bach. This is accompanied by a syntactic tree depicting the hierarchical patterning of tension and relaxation motions embedded in larger scale motions. (from Patel, 2003).

In the music domain, discrete acoustic sounds are grouped into a set and assembled with other sets according to certain rules “beyond their temporal order” (Thompson-Schill et al., 2013). This rule-based arrangement of musical sets is defined as musical syntax endowed with the property of hierarchical processing as in the language domain (Figure 3; Patel, 2003, 2010). Hierarchical processing in music has been studied mainly in the classical theory of harmony, that is, certain regularities about the arrangement of chord functions within harmonic sequences which express complex long-distance hierarchical relationships between musical events (Rohrmeier, 2011; Koelsch, 2013). For example, a chord sequence was used in a number of fMRI studies to generate strong or weak expectancy for harmonically related or unrelated chords, leading to the building of a musical syntax (Koelsch, 2011), and bilateral BA44, with right hemisphere weighting, was found to be involved in this process (Koelsch et al., 2002, 2005; Tillmann et al., 2003; Koelsch and Siebel, 2005).

With respect to the hierarchical aspect of music and its relation to language, “shared syntactic integration resource hypothesis” is often addressed to explain the interaction between musical and linguistic syntax; the neural substrates and computations involved in the processing of linguistic syntax can be shared with those involved in the processing of musical structure (Patel, 2003). Along with the convergence of language and music, evidence for the overlap between the two domains has also been found using electrophysiological measures. One of the ERP studies showed that a common P600 component known to be sensitive to syntactic processing was observed when participants judged out-of-key chords (harmonically unexpected chords) in music, which suggested that the neural mechanism for the processing of hierarchical structure was similar between the music and language domains (Patel et al., 1998). Another study using MEG showed the magnetic equivalent of an early right-hemispheric anterior negativity (mERAN) for music, resembling the early left anterior negativity (ELAN) for language when participants listened to an out-of-key chord (Maess et al., 2001). This was evoked by hierarchical dependencies as an indicator of early syntactic processing in music (Koelsch et al., 2013) and more interestingly the source of the mERAN was localized in Broca’s area and its right homolog. In a recent ERP study where musicians imitated silent videos of a right hand playing congruent or incongruent sequences of chords, a consistent finding was reported that ERAN was evoked when musicians observed and reprogrammed the playing of incongruent chords (Sammler et al., 2013).

Intriguingly, some studies had an experimental design where participants were divided into two groups (i.e., experts vs. non-experts in music) to investigate the influence of musical proficiency in the processing of musical syntax. In a near-infrared spectroscopy study by Wakita (2013), two groups of participants (well-trained and less-trained groups in music) watched silent movies of hierarchically organized hand movements playing familiar and unfamiliar melodies. The results discovered increased activation in Broca’s area in the unfamiliar melody condition, but, more interestingly, only in the well-trained group. This fits with the result of Sammler et al. (2013) mentioned above where they also recruited pianists who had a minimum of 14 years of musical training. The difference between high- and low-proficient groups in the process of musical hierarchy addresses the issue of the level of proficiency and its potential influence on hierarchical processing, which should be further investigated in the future.

## Hierarchical processing in mental arithmetic

Whether hierarchical computation in language and mathematic formula depends on a common neural mechanism or not has been a controversial issue. It has been claimed that the human faculty for arithmetical reasoning is abstracted from the computation of language so that the number faculty is developed as a “by-product of the language faculty” (Chomsky, 1988; Hauser et al., 2002; Fitch et al., 2005). On the contrary, some studies, mostly from brain-damaged patients, insisted that the two processes are independent of each other such that patients with preserved mathematical skills showed severe impairment in language processing (Cappelletti et al., 2001; Varley et al., 2005), or vice versa (Lucchelli and De Renzi, 1993; Dehaene and Cohen, 1997).

**Figure 4:**
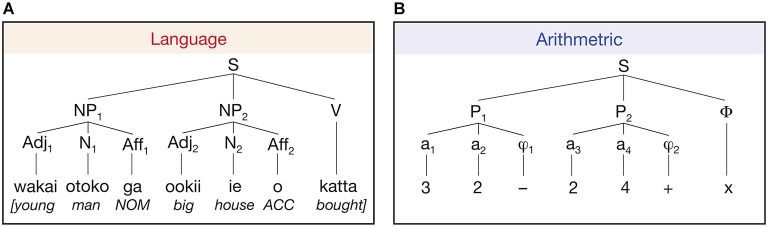
**Two-level build-up of hierarchical structures in language (Japanese) and arithmetics (Polish notation). (A)** In Japanese, case-marking particles (“ga” for nominative and “o” for accusative) are positioned to the right of the noun and the verb is located at the end of the sentence. NP, noun phrase; Adj, adjective; N, noun; Aff, affix (i.e., case-marking particle); V, verb; NOM, nominative case; ACC, accusative case. **(B)** In reverse Polish notation, two natural numbers precede binary arithmetic operators. The example denotes (3 − 2) × (2 + 4). In both language and arithmetics, similar hierarchical processing is required in that computation with the case markers or arithmetic operators follows the two elements (words or numbers). (from Makuuchi et al., 2012).

A number of neuroimaging studies investigated the role of language in the process of arithmetic. For example, Makuuchi et al. (2012) investigated the process of building up a hierarchical structure in language and arithmetic with respect to combining individual elements (i.e., words or numbers) following certain rules and orders (i.e., case markers or arithmetical operators). Interestingly, they used a reverse Polish notation for the arithmetical domain so that hierarchical processing between the two domains was equated (Figure 4). They found a significant activation in the dorsal part of the pars opercularis (BA 44) in processing a complex hierarchy in both domains, suggesting a domain-general characteristic of this area. On the other hand, Maruyama et al. (2012), setting up four levels of hierarchies in the arithmetic calculation with +/− operators and parentheses, argued that hierarchical processing in arithmetic is “compiled” in bilateral ventral occipito-temporal cortices known as the visual word form area. Particularly noteworthy is the fact that they, dissimilar to Makuuchi’s experiment, recruited well-trained participants with a high level of proficiency in mathematics and in whom simple arithmetic calculation may be routinized. Therefore, the level of proficiency might be the main factor for the opposing results between the two studies. As already mentioned in the previous section where experts and non-experts in musical hierarchy showed disparate patterns of brain activation, the issue regarding the level of proficiency should not go unheeded and should be further investigated in connection with hierarchical processing in general.

## Hierarchical processing in actions

Here, the discussion about hierarchical processing is expanded to the action domain in the sense that complex human behavior is also hierarchically composed of simple motor controls according to a set of rules (“motor syntax”) to achieve temporally distal goals (Grafton and Hamilton, 2007), which is in line with the definition of hierarchical processing as defined in the Introduction. According to Cooper et al. (2014), one of the major characteristics of human actions is that they are goal-directed and hierarchically structured. The PFC, more specifically Broca’s area, has been known to be engaged in the goal-directed action domain. In the neuropsychological study of Fazio et al. (2009), aphasic patients without apraxic symptoms who had higher lesion overlaps in the pars opercularis (BA 44) performed a task of organizing the four video-snapshots of either human actions or physical events. A clear dissociation was observed in their performance which showed impairment in human actions but not in physical events. A virtual lesion study with TMS also showed that stimulating over left BA44 in normal participants resulted in more impairment for organizing biological actions (i.e., human actions) compared to nonbiological actions (i.e., object in movement) (Clerget et al., 2009). These two studies focused on encoding human action where participants were expected to understand the general goal of the action and correctly reorder simple motor acts based on a certain motor hierarchy, showing the vital role of BA44 in the hierarchical processing of an action.

**Figure 5:**
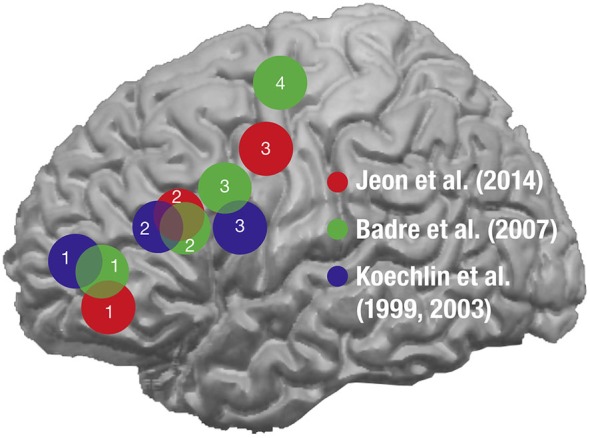
**A schematic display of the approximate distribution of the activation foci depending on the levels of cognitive controls in various studies**. The approximate distribution of the peak activations is displayed with spheres from the studies where the functional subdivisions of the PFC were investigated depending on the levels of hierarchy in the cognitive controls: Jeon et al. (2014) in red, Badre and D’Esposito (2007) in green, and Koechlin et al. (1999), (2003) in blue. The numbers denoted within the activations indicate the hierarchy with 1 for the highest level and 4 for the lowest level. The posterior-to-anterior patterns of activations were observed as the level of hierarchy became higher across all the studies. (from Jeon et al., 2014).

The notion of action hierarchy can be extended to *cognitive control*, which refers to the ability to coordinate or guide thoughts or actions in relation to internal representations of goals, plans, and context (Badre, 2008). Several theories have been suggested to explain the possible framework for generating different levels of hierarchies in the cognitive controls within the PFC (for review, see Koechlin and Summerfield, 2007; Badre, 2008; Botvinick, 2008). It has been proposed that cognitive control is hierarchically organized and a posterior-to-anterior axis of the PFC is functionally subdivided depending on the different levels of processing hierarchies of the cognitive controls (Figure 5). One of the noteworthy theories for ranking the levels of hierarchy is *temporal abstraction*, which suggests that a significant fraction of cognitive controls is based on the temporal framing and the context (Koechlin and Summerfield, 2007, 2008). According to this theory, the multi-stage system of cognitive controls can be categorized into three levels of increasing ranking order: contextual, episodic, and branching controls. It has been suggested that these three levels of cognitive control form a cascade of top-down selective processes operating along the rostrocaudal axis (also known as the posterior-to-anterior axis) of the lateral PFC, with the contextual, episodic, and the branching controls being subserved in the posterior lateral PFC (BA44), the anterior lateral PFC (BA45), and the frontopolar lateral PFC (BA10), respectively (Koechlin et al., 1999, 2003; Koechlin and Jubault, 2006; Koechlin and Summerfield, 2007; Jeon et al., 2014). Therefore, Broca’s area, being implemented within the posterior-to-anterior network and involved relatively in the lower levels of hierarchy, may also be associated with the processing of hierarchical structures of the cognitive controls, further reinforcing the supramodal involvement of BA44 for hierarchical processing in human cognition (Koechlin and Jubault, 2006).

## Conclusion

Since early 1950 when Karl Lashley argued that human behavior displayed hierarchical structure comprising nested subroutines, many studies have been conducted on hierarchical models of behavior (Lashley, 1951). The essential feature of hierarhical processing is to mediate temporally remote as well as adjacent processses to perform a wide range of cognitive and motor activities successfully. Therefore, discussing hierarchical processing is always accompanied by the concept of time, which inevitably necessitates the binding role of the PFC in terms of cross-temporal contingencies. Along with the birth of computational modeling and the development of neuroimaging techniques, the neural mechanisms underlying hierarchical processing have been scrutinized, being accumulated with ample evidence of the involvement of the PFC.

In line with this, this review discussed hierarchical processing with respect to its neural substrates within the PFC. Evidence has been adduced to explain that hierarchical processing can be actively conducted in various cognitive domains including language, visuo-spatial sequence, music, mathematics, and action. Across the domains, BA44 seems to be the critical subregion of the PFC which has been repeatedly observed when the ongoing cognitive tasks are associated with hierarchical processing. However, as already discussed in the section on music and mental arithmetic domains, the issue of the level of proficiency and its influence on the processing of cognitive hierarchies should be further investigated. In order to do this, having two groups of participants, that is, groups with a high level and low level of proficiency, will be an important configuration.

All the processes mentioned in this review discuss the hierarchical processing with symbols; language is inherently a symbolic process; visual symbols were used for visuo-spatial sequences; musical notes were recruited for musical hierarchy; numbers and arithmetical operators were used as mathematical symbols. This brings about an interesting question: Is it possible to conceptualize hierarhical processing without symbols, that is, non-symbolic hierarchical processing? This may be an interesting topic for future study.

Finally, we should not lose sight of the fact that cognitive processes are made up of neural transactions within and between several brain regions as well as networks. The cognitive function of the PFC is mediated together with orbitomedial and posterior association cortices, the striatum, and other subcortical structures through cortico-striatal-thalamo-cortical loops which are topographically organized and functionally segregated in each loop (Alexander et al., 1986; Grahn et al., 2008; Jeon et al., 2014). Therefore, large-scale functional specificity and structural connectivity pertaining to different levels of cognitive hierarchies should be addressed in the subcortical areas such as the striatum or thalamus as well as the PFC.

## Conflict of interest statement

The author declares that the research was conducted in the absence of any commercial or financial relationships that could be construed as a potential conflict of interest.
